# Modulating gut dysbiosis and mitochondrial dysfunction in oxazolone-induced ulcerative colitis: the restorative effects of β-glucan and/or celastrol

**DOI:** 10.1080/13510002.2022.2046425

**Published:** 2022-03-04

**Authors:** Omnia Safwat El-Deeb, Rasha Osama El-Esawy, Hanan Alsaeid Al-Shenawy, Heba Bassiony Ghanem

**Affiliations:** a Medical Biochemistry Department, Faculty of Medicine, Tanta University, Tanta, Egypt; b Pharmacology Department, Faculty of Medicine, Tanta University, Tanta, Egypt; c Pathology Department, Faculty of Medicine, Tanta University, Tanta, Egypt; d Clinical Laboratory Sciences Department, College of Applied Medical Sciences, Jouf University, Sakaka, Saudi Arabia

**Keywords:** Ulcerative Colitis, β-Glucan, Celastrol, TMAO, FFAR2, PGC-1α, Dysbiosis, ROS

## Abstract

**Objectives:**

Microbiome–Mitochondria interaction is gaining a significant attention; thus, studying its mechanism emerges as a must to provide restorative lines in managing diseases. The aim is to study the mechanistic effects of β-Glucan and/or Celastrol in oxazolone-induced ulcerative colitis (UC).

**Methods:**

75 Wistar rats were allocated into 5 equal groups. Group I: control group. Group II: UC group, Group III: β-Glucan-treated UC group, Group IV: Celastrol-treated UC group & Group V: mutual treatment group. All groups were subjected to the detection of free fatty acid receptor 2 (FFAR-2) and peroxisome proliferator-activated receptor gamma co-activator1α (PGC-1α) mRNA gene expressions. Citrate synthase (CS) activity, mitochondrial membrane potential (MMP), ATP concentration, reactive oxygen species (ROS) were detected. Trimethylamine N-oxide (TMAO) concentration was measured.

**Results:**

After treatment we monitored significant upregulation of FFAR-2 and PGC-1α mRNA expression. Likewise, ATP level and CS activity were significantly increased. On the contrary, there was a significant lessening in ROS and TMAO levels with improvement of MMP.

**Conclusion:**

Mutual use of β- Glucan and Celastrol had a greater effect than each alone against UC, which is considered a novel finding highlighting the ameliorative effects of this combined treatment in modulating Microbiome/Mitochondria axis, thus launching promising avenues for UC.

## 1. Introduction

Inflammatory bowel diseases (IBDs) are a group of chronic long-standing inflammatory disorders with a lingering relapsing course characterized by inflammation of the intestinal mucosa [1]. Among these disorders, ulcerative Colitis (UC) is the most predominant IBD known one. Of note, the pathogenesis of these diseases is driven by many factors comprising deregulated immune responses, genetic predisposition, commensal microbiota disruption, and epithelial barrier defects [2]. Furthermore, massive bacterial overload initiates an acute inflammatory intestinal response if the mucosal barrier is broken and bacteria gain access to the lamina propria. UC is represented clinically with distinctive symptoms, e.g. abdominal pain, weight loss, diarrhea and bleeding per rectum [3].

The microorganisms within the body with diverse physio-chemical properties, demarcated as the microbiome, have a far-reaching impact on the usual physiology and is a metabolically active endogenous ‘organ’ by itself with metabolic capacity beyond that of the liver [4]. The microbiota is a critical element of the immune system, gut-related lymphoid tissue; thus turbulences to it might cause various diseases. Previous research studies acknowledged fluctuations in gut microbiota balance in UC patients, highlighting the imminent role of microbial flora oscillations in UC pathogenesis [5,6].

Owing to their capability to yield ATP via respiration, mitochondria became crucial organelles in preserving cell function and homeostasis. The incorporation of the mitochondria's numerous roles in cellular function together with mitochondrial dysfunction participation into disease pathogenesis has recently come into the focus. Thus, recent studies have recommended an intimate association between mitochondrial dysfunction and UC, but the precise mechanism is still indistinctive [7]. Moreover, that mitochondria are the most responsive organelle to microbiotic signaling, as the microbiome produces a broad spectrum of small metabolites that can intensely influence mitochondrial homeostasis [8].

The specific group of polysaccharides composed of D-glucose monomers linked by β-glycosidic bonds, defined as β-Glucan, is present in the plants’ cell wall, fungi and bacteria as the main constituents providing structural support [9]. β-glucans’ fermentation by microbes in the end part of the small intestine results in short-chain fatty acids (SCFAs) [10] that activate free fatty acid receptors 2 and 3 FFAR2 and 3. They obtain numerous positive effects on GIT and systemic health [11]. For instance, they lessen the gut pH, helping the reticence of pathogenic microorganism evolution [12].

The dienone-phenolic triterpene, or Celastrol, is the biochemically active component extracted from Tripterygium wilfordii (TW), a customary Chinese therapeutic herb [13,14]. Because of remedial properties, it is used in treating many disorders, such as neurodegenerative, inflammatory and auto-immune diseases, by modulating a variability of molecular targets. Moreover, it causes colon injury and reduced its severity, recovering intestinal homeostasis in an induced UC mice model [15].

### 1.1. Aim of the study

The ultimate remedy for UC has not yet been recognized, and the available treatments are limited to the preservation of remission. In this setting, our research study aimed to highlight the role of β-Glucan and/or Celastrol in modulating the gut microbiome-mitochondrial axis in managing UC as an acute autoimmune disease with distressing medical & socioeconomic burden.

## 2. Material and methods

### 2.1. Chemicals

Oxazolone (4-Ethoxymethylene-2-phenyl-2-oxazolin-5-one, CAS NO: 15646-46-5), Celastrol (≥98 purity, CAS NO: 34157-83-0) and other chemicals and solvents used, unless otherwise described, were purchased from Sigma (Sigma, St Louis, MO, USA), while β-Glucan was purchased from EUSA colors (ASIA) limited; Tangshan, Hebei, China. All chemicals and solvents were of high analytic grade.

### 2.2. Animals

Animals were obtained from the Animal House, Biochemistry Department, Faculty of Medicine, Tanta University. They were 75 male Wistar albino rats; their weight ranged from 180 to 200 g. They were housed in standard rat cages in groups of five per cage. They were allowed food and water ad libitum. Constituents of the experimental diet (g/kg diet) were according to the formula of Kim et al. [16]. It included the normal diet for control rats (Fat 5% [corn oil 5%], carbohydrates 65% [corn starch 15% and sucrose 50%], proteins 20.3% [casein 20% and DL-Methionine 3%], fiber 5%, salt mixture 3.7%, and vitamin mixture 1%). For one week prior to the experiments, animals were acclimatized to these conditions. Experimental protocols and animal handling were performed according to the Ethical Committee of the Faculty of Medicine, Tanta University, and they comply with the Guide for the Care and Use of Laboratory Animals (Institute of Laboratory Animal Resources, 1996). All the experiments were performed in the Biochemistry Department, Faculty of Medicine, Tanta University.

### 2.3. Experimental design

Induction of UC was achieved, as earlier described by Boirivant et al. [17]. Briefly, oxazolone solution was prepared by dissolving in 40% (v/v) aqueous ethanol to a final concentration of 7.5 mg/mL and given once in a dose of 1.1 mL/rat into the colon via a rubber catheter introduced 4 cm inside the anal canal under light anesthesia. Later, we removed the catheter, and the rat was held vertically for 30 s to make sure that the oxazolone was distributed properly within the whole colon and cecum.

The rats were allocated into equal 5 groups (each of 15 rats) as follows:

**Group I** (the control group): given 40% aqueous ethanol once in a dose of 1.1 mL/rat into the colon via a rubber catheter under light ether anesthesia.

**Group II** (the UC group): presented to be the untreated group with oxazolone-induced colitis.

**Group III** (the β-Glucan-treated group): oxazolone-induced colitis with concomitant administration of β-Glucan by intragastric gavage in a dose of 350 mg/kg/day (at a concentration of 140 mg/mL in distilled water) for 21 days [18].

**Group IV** (the Celastrol-treated group): oxazolone-induced colitis with associated administration of Celastrol by intraperitoneal injection in a dose of 1 mg/kg/day (at a concentration of 1 mg/mL solution in 1% DMSO) for 21 days [19].

**Group V** (the combined β-Glucan and Celastrol-treated group): oxazolone-induced colitis with the associated administration of β-Glucan and Celastrol in the same doses and duration as mentioned previously.

### 2.4. Tissue sampling and homogenate preparation

All rats were sacrificed by decapitation under 2–5% isoflurane anesthesia. Intestinal tissues of rats were split, weighed and divided into three parts: For histopathological and immunohistochemical studies, one part was preserved in buffered formaldehyde (10%), For the second part, tissue homogenates and separated of mitochondria and cytosol; mitochondria and cytosol were sequestered by differential centrifugation as formerly described [20]. The isolation of mitochondria was performed; in brief, cells were collected and mixed up in a buffer containing 250 mM sucrose, 0.5 mM EGTA, 0.5 mM EDTA, 3 mM HEPES-NaOH, protease and phosphatase inhibitor cocktails (with adjusted pH 7.2). The mixture was homogenized on ice for 1 min and centrifuged at 1000*g* for 10 min at 4°C. To obtain a pure mitochondrial lysate, the supernatant was carefully removed, and the pellet was centrifuged again at 12,000*g* for 15 min at 4°C. The secondary supernatant was discarded, and the pellet containing mitochondria was re-suspended till further use [20]. The tissue was scissor chopped and homogenized on ice. Tissue homogenate was centrifuged at 1000*g* for 5 min at 4°C in 1.5 mL Eppendorf tubes to pellet cell fragments, and the supernatant was partially frozen at 80°C until further use for biochemical markers, while the remaining half was centrifuged at 9500 *g* for 10 min to pellet nuclei. The mitochondrial fraction and the soluble cytosolic fraction were obtained by centrifuging the supernatant at 14,000*g* for 25 min. Tissues protein concentrations were determined by the Lowry method [21]. Finally, for gene expression analysis, the third part of tissues was stored at −80°C till used.

### 2.5. Biochemical assays

#### 2.5.1. Detection of the mitochondrial parameters

##### 2.5.1.1. Mitochondrial ATP concentration

Mitochondrial ATP levels were estimated using colorimetric ATP assay kits (Cat# E-BC-K157-S) supplied by Elabscience Company, Houston, TX, USA. The absorbance of each sample was read at 636 nm in a semiautomatic BTS-350 Biosystems spectrophotometer.

##### 2.5.1.2. Mitochondrial citrate synthase (CS) activity assay

CS activity (EC 4.1.3.7) in isolated mitochondria was assessed as previously described [22]. After thawing of mitochondrial extract samples, a reaction solution, containing 0.1 mM dithionitro benzoic acid (DTNB), 0.5 mM oxaloacetate, 50 mM EDTA, 0.31 mM acetyl coenzyme A, 5 mM triethanolamine hydrochloride, and 0.1 M Tris–HCl, was mixed and preheated for 5 min at 30°C. Following that, the mitochondrial extract was added to the reaction medium, and CS activity was measured spectrophotometrically at 412 nm and expressed as micromole per minute per milligram of protein.

##### 2.5.1.3. Mitochondrial transmembrane potential (ΔΨm) assay

MMP was estimated following by the Maity et al.’s method [23]. The isolated mitochondria (20 μg) were incubated with JC-1 (300 nm) in the dark for 10 min at 37°C in JC-1 assay buffer. The fluorescence of each sample was measured in a spectrofluorometer (excitation, 490 nm; slit, 5 nm; emission, 590 nm for J-aggregate and 530 nm for J-monomer; slit, 7.2 nm).

##### 2.5.1.4. Mitochondria reactive oxygen species (ROS) assay

It was assessed spectrophotometrically using the instruction manual of mitochondrial ROS assay kits (Cat# No. 701600) supplied by Cayman Chemical Company, Ellsworth Ann Arbor, MI, USA. Positive control was used with two replicates. We added Antimycin A in positive control wells using a maximum concentration 10 uM. Complex III binds Antimycin A to a domain of cytochrome bH, which inhibits cytochrome bc1 (complex III) and blocks electron transport from the heme bH center to ubiquinone. This inhibition of mitochondrial electron transport also markedly increased mitochondrial ROS formation.

#### 2.5.3. Detection of microbiota-derived metabolite; Trimethylamine N-oxide (TMAO) level

Tissue TMAO level was detected according to the Wekell method [24] using an equimolar mixture of ferrous sulfate and disodium EDTA. The absorbance of each sample was read at 410 nm in a semiautomatic BTS-350 Biosystems spectrophotometer using the sample blank for reference.

#### 2.5.4. Quantitative measurement of colonic Free fatty acid receptor 2 (FFAR-2) and Peroxisome proliferator-activated receptor-γ coactivator-1α (PGC-1α) mRNA expression by quantitative real-time reverse transcription PCR (RT–PCR)

- Gene JET RNA Purification Kit, following the manufacturer’s directions, was used to separate total RNA (#K0731, Thermo Scientific, Waltham, MA, USA). Revert Aid H Minus Reverse Transcriptase (#EP0451, Thermo Scientific, USA) was used to reverse-transcribed 5 μg of total RNA to obtain cDNA. Applied Biosystem, Step One Plus real-time PCR system (USA), was used to detect the relative expression of the CNTF gene using the cDNA as a template.
- The primers were designed by software (Primer 5.0) and their sequences specific for rat were as follows:

**Table:** 

Gene	Forward	Reverse	Accession number
FFAR-2	5′ CTACGAGAACTTC ACCCAAGAG 3′	5′ GAAGCGCCAATAACAGAAGATG 3′	XM_039104536.1
PGC-1α	5′ GTGCAGCCAAGACTCTGTATGG 3`	5′ GTCCAGGTCATTCACATCAAGTTC3`	XM_039092489.1
GADPH	5’ CAACTCCCTCAAGATTGTCAGCAA 3'	5’ GGCATGGACTGTGGTCATGA 3'	NM_001394060.2

- 2X Maxima SYBR Green/ROX qPCR Master Mix (12.5 μL) were purchased from Thermo Scientific, USA (Catalog# K0221); cDNA template (2 μL), forward primer (1 μL), reverse primer (1 μL) and nuclease-free water (8.5 μL) were added to organize a 25-μL PCR mix. The following circumstances were those of thermal cycling: 95°C for 10 min for initial denaturation, 40 cycles of amplification of DNA denaturation at 95°C for 15 s, 60°C for 30 s for annealing, 72°C for 30 s for an extension. The temperature was increased from 63 to 95°C at the last cycle ending for melting curve analysis.

The comparative cycle threshold **^ΔΔ^** Ct method was used to detect the relative levels of gene expression, which was normalized to the housekeeping gene [25]

### 2.5. Histopathological preparation and evaluation of the groups

After successful modeling, the lesions of mice were fixed with 10% formaldehyde and embedded with paraffin to prepare paraffin sections. The sections were stained with hematoxylin and eosin to confirm the diagnosis and tissue damage assessment based on a simplified Geboes scoring system [26]. The morphology of sections was observed under an optical microscope and tissue damage index (TDI).

### 2.6. Immunohistochemical study and assessment of the studied groups

Sections from formalin-fixed specimens embedded in paraffin were attached on positively charged slides then autoclaved at 58°C for 24 h. Deparaffinized and rehydrated sections were engrossed in 10 mL/L H2O2 for 20 min, and then they were washed 3 times, each time for 3 min with PBS. Sections were preincubated with 10 mL/L normal goat anti-rabbit serum for 20 min at RT and then were incubated with the antibodies diluted for 18 h at 4°C and Envision reagent for 30 min at 37°C. Sections were stained by 0.4 g/L DAB with 0.3 mL/L H_2_O_2_ for 8 min and hematoxylin for 30 s. The results were observed under a light microscope. PBS instead of the primary antibody was used as the negative control.

Antibodies used were Bcl2 (clone 124, code no. M0887, IgG1, kappa; Dako, Glostrup, Denmark) and Bax (IgG; Oncogene Science, Inc, USA). Immunohistochemical staining assessment was achieved using a light microscopy (10× objective lens) with the selective use of a 20–40×objective lens for validation. Immunoreactivity interpretation was accomplished semantically by investigating the staining cytoplasmic positivity extent. Immunostaining of less than 10% of epithelial cells was scored +1; 10–50% positivity scored +2; 51–90% scored +3; while >90% positivity scored +4 [27].

### 2.7. Statistical analysis

The whole data were presented as means ± SD. Using one-way ANOVA, the statistical significance was projected using SPSS 18.0 software. *P*-values were deliberated statistically significant when *P*≤ 0.05. Tukey’s Honestly Significant Difference (Tukey’s HSD) test was performed to compare the means.

## 3 . Results

### 3.1. Effect of β-glucan and Celastrol on colonic TMAO

Table 1 shows a significant escalation in colonic TMAO in the UC group (Group II) compared to the control (Group I) and other treated groups (Groups III, IV and V). However, the combined β-glucan and Celastrol-treated group (Group V) displayed a significant decrease in colonic TMAO level compared to the UC (Group II) and other treated groups (Groups III and IV). Meanwhile, the β-glucan-treated group (Group III) showed a significant decrease in colonic TMAO level compared to the Celastrol-treated group (Group IV).

**Table 1.** Effect of β-glucan and celastrol on different parameters in the studied groups.

Parameter/Groups	Group I (*n* = 15)	Group II (*n* = 15)	Group III (*n* = 15)	Group IV (*n* = 15)	Group V (*n* = 15)	*F*-value	*P*-value
TMAO (pmol/mg protein)	13.5 ± 2.6	122.8 ± 5.6^a^	67.8 ± 9.2^a,b^	76.5 ± 9.3^a,b,c^	51.5 ± 10.1^a,b,c,d^	379.5	<.001*
Mitochondrial ATP Concentration (nmol/mg protein)	13.7 ± 2.1	5.7 ± 1.4^a^	7.6 ± 0.99^a,b^	9.7 ± 079^a,b,c^	11.3 ± 1.6^a,b,c,d^	59.22	<.001*
Mitochondrial citrate synthase activity (IU/mL)	9.67 ± 1.6	2.1 ± 1.1^a^	4.9 ± 1.03^a,b^	6.2 ± 0.9^a,b,c^	7.4 ± 1.06^a,b,c,d^	90.23	<.001*
Mitochondrial Membrane Potential (ΔΨm) (Florescent Unit)	8.13 ± 1.1	2.4 ± 0.9^a^	4.5 ± 0.8^a,b^	5.9 ± 0.7^a,b,c^	7 ± 1.1^a,b,c,d^	86.92	<.001*

Notes: Values are expressed as mean ± SD. The number of rats in each group (*n* = 15). *P* was considered significant at <.05. a, Significance vs. Group I; b, Significance vs. Group II, c, Significance vs. Group III and d, Significance vs. Group IV, using one-way ANOVA followed by Tukey’s *post hoc* test for multiple comparisons.

### 3.2. Effect of β-glucan and Celastrol on mitochondrial ATP concentration, membrane potential (ΔΨm), reactive oxygen species (ROS) and CS activity

Table 1 demonstrates a significant reduction in mitochondrial ATP concentration, MMP and CS activity in the UC group (Group II) compared to the control (Group I) and other treated groups (Groups III, IV and V). Additionally, the combined β-glucan and Celastrol-treated group (Group V) displayed a significant escalation in mitochondrial ATP concentration, MMP and CS activity compared to the UC (Group II) and other treated groups (Groups III and IV). Additionally, the Celastrol-treated group (Group IV) showed a significant escalation in mitochondrial ATP concentration, MMP and CS activity compared to the β-glucan-treated group (Group III).

On the other hand, there was a significant increment in mitochondrial ROS in the UC group (Group II) compared to the control (Group I) and other treated groups (Groups III, IV and V). Furthermore, the combined β-glucan and Celastrol-treated group (Group V) displayed a significant decrease in mitochondrial ROS compared to the UC (Group II) and other treated groups (Groups III and IV). Moreover, the Celastrol-treated group (Group IV) showed a significant decrease in mitochondrial ROS compared to the β-glucan-treated group (Group III), as shown in Table 2.

**Table 2.** Effect of β-glucan and celastrol on mitochondrial reactive oxygen species in the studied groups.

Parameter/Groups	Group I (n = 15)	Group II (n = 15)	Group III (n = 15)	Group IV (n = 15)	Group V (n = 15)	Positive Control	F-value	*P*-value
Mitochondrial Reactive Oxygen Species (pmol/min/mg protein)	5.3 ± 0.98	20.4 ± 1.2^a^	15.7 ± 1.2^a,b^	13.13 ± 1.1^a,b,c^	9.8 ± 0.78^a,b,c,d^	25.56 ± 0.77^a,b,c,d,e^	413.6	<0.001*

Notes: Values are expressed as mean ± SD. The number of rats in each group (*n* = 15). *P* was considered significant at <.05. a, Significance vs. Group I; b, Significance vs. Group II; c, Significance vs. Group III; d, Significance vs. Group IV and e, Significance vs. Group V using one-way ANOVA followed by Tukey’s *post hoc* test for multiple comparisons.

Table 3 demonstrates significant negative correlations of colonic TMAO with mitochondrial ATP concentration, MMP and mitochondrial CS activity in the UC-diseased group (Group II) and treated groups (Group II, IV and V). In addition, there were negative correlations of mitochondrial ROS with mitochondrial ATP concentration, MMP and mitochondrial CS activity in the UC-diseased group (Group II) and treated groups (Group II, IV and& V), as shown in Table 3. Furthermore, Table 3 shows positive correlations between mitochondrial ATP concentration, MMP and mitochondrial CS activity and positive correlations between TMAO and mitochondrial ROS in the UC-diseased group (Group II) and treated groups (Groups II, IV and V).

**Table 3.** Correlation between different studied parameters among the different studied groups.

	TMAO (pmol/mg protein)		Mitochondrial ATP Concentration (nmol/mg protein)		Mitochondrial citrate synthase activity (IU/mL)		Mitochondrial Membrane Potontial (ΔΨm) (Florescent Unit)		Mitochondrial reactive oxygen species (pmol/min/mg protein)	
	*r*	*P*	*r*	*P*	*r*	*P*	*r*	*P*	*r*	*P*
TMAO (pmol/mg protein)	–	–	−0. 64	<.001*	−0.79	<.001*	−0.78	<.001*	0.85	<.001*
Mitochondrial ATP Concentration (nmol/mg protein)	−0. 64	<.001*	–	–	0. 72	<.001*	0.78	<.001*	−0.81	<.001*
Mitochondrial citrate synthase activity (IU/ml)	−0.79	<.001*	0. 72	<.001*		–	0.81	<.001*	−0.86	<.001*
Mitochondrial Membrane Potontial (ΔΨm) (Florescent Unit)	−0.78	<.001*	0.78	<.001*	0.81	<.001*	–	–	−0.85	<.001*
Mitochondrial reactive oxygen species (pmol/min/mg protein)	0.85	<.001*	−0.81	<.001*	−0.86	<.001*	−0.85	<.001*	–	–

*P < 0.05.

### 3.3. Effect of β-glucan and Celastrol on colonic PGC-1α gene expression

**Figure 1.**
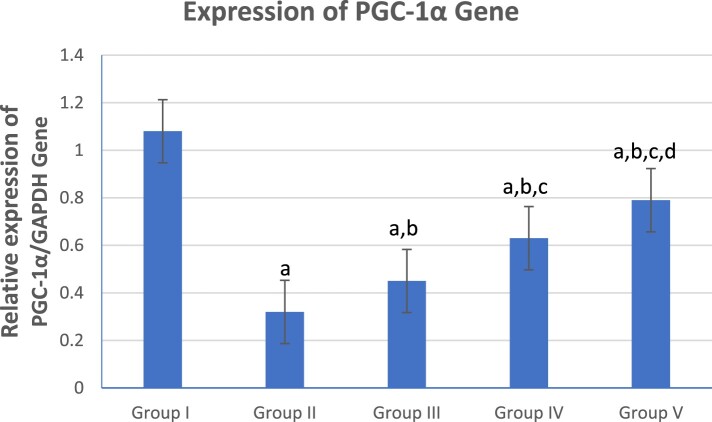
Graphical presentation of real-time quantitative PCR analysis of the expression of PGC-1α gene in studied groups by fold change. Means within column carrying different superscript letters are significantly different at *p* ≤ 0.05; a, significance vs. Group I; b, significance vs. Group II; c, significance vs. Group III; d, significance vs. Group IV.

Figure 1 shows a graphical presentation of real-time quantitative PCR analysis of the expression of the PGC-1α gene in studied groups by fold change. There was a significant reduction in colonic PGC-1α gene expression in the UC group (Group II) compared to the control (Group I) and treated groups (Groups III, IV and V). However, the combined β-glucan and Celastrol-treated group (Group V) displayed a significant escalation in colonic PGC-1α gene expression compared to the UC (Group II) and other treated groups (Groups III and IV).

Meanwhile, the Celastrol-treated group (Group IV) showed a significant escalation in colonic PGC-1α gene expression compared to the β-glucan-treated group (Group III).

### 3.4. Effect of β-glucan and celasterol on colonic free fatty acid receptor-2 (FFAR-2) gene expression

**Figure 2.**
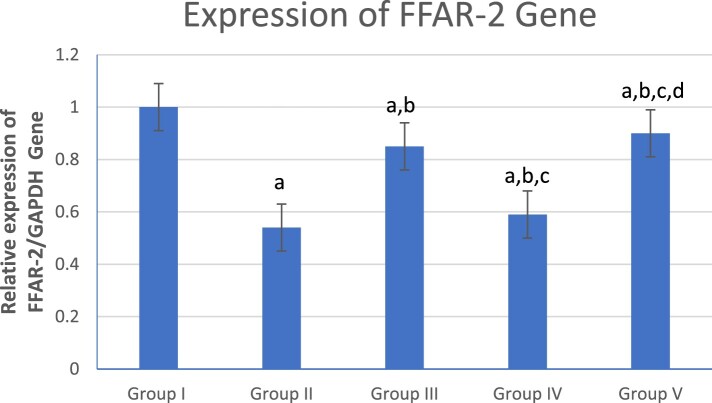
Graphical presentation of real-time quantitative PCR analysis of the expression of FFAR-2 gene in studied groups by fold change. Means within column carrying different superscript letters are significantly different at *p* ≤ 0.05; a, significance vs. Group I; b, significance vs. Group II; c, significance vs. Group III; d, significance vs. Group IV.

Figure 2 shows a significant decrease in colonic FFAR-2 gene expression in the UC group (Group II) compared to the control (Group I) and other treated groups (Groups III, IV and V). However, the combined β-glucan and Celastrol-treated group (Group V) displayed a significant increase in colonic FFAR-2 gene expression compared to other UC (Group II) and treated groups (Groups III and IV). Meanwhile, the β-glucan-treated group (Group III) showed a significant increase in colonic FFAR-2 gene expression compared to the Celastrol-treated group (Group IV).

### 3.5 Histopathology and immunohistochemical of the studied groups

**Figure 3.**
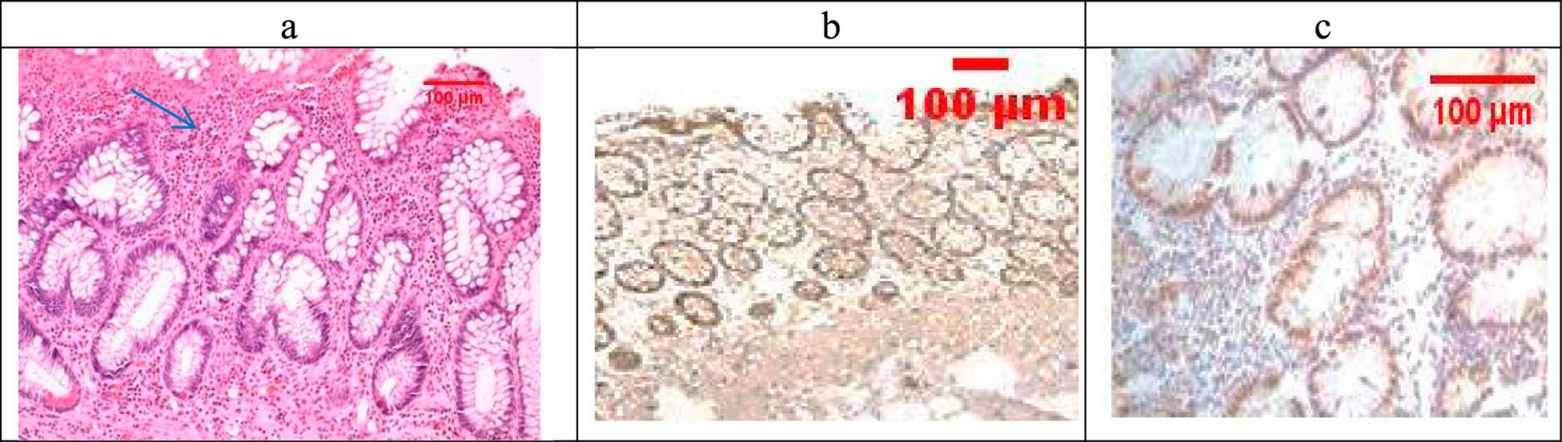
(a) Ulcerative colitis Geboes score 2B.2 showed a marked increase in the neutrophils in the lamina propria (blue arrow, H&E ×100), (b) the same case showed score 3 Bcl-2 expressions (ABC ×100), (c) also showed score 2 expressions of Bax (ABC ×200).

**Figure 4.**
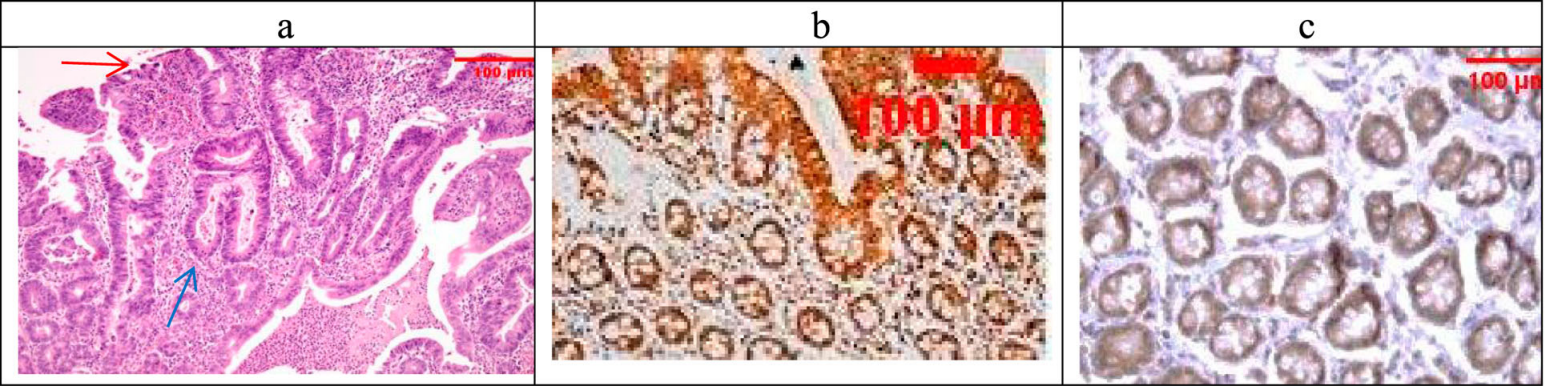
(a) A case of UC Geboes score 4.4 showed marked epithelial injury, cryptitis (blue arrow) and ulcerations (red arrow) (H&E ×100), (b) the same case showed score 4 Bcl2 expressions (ABC ×100), (c) also showed score 4 expressions of Bax (ABC ×100).

**Figure 5.**
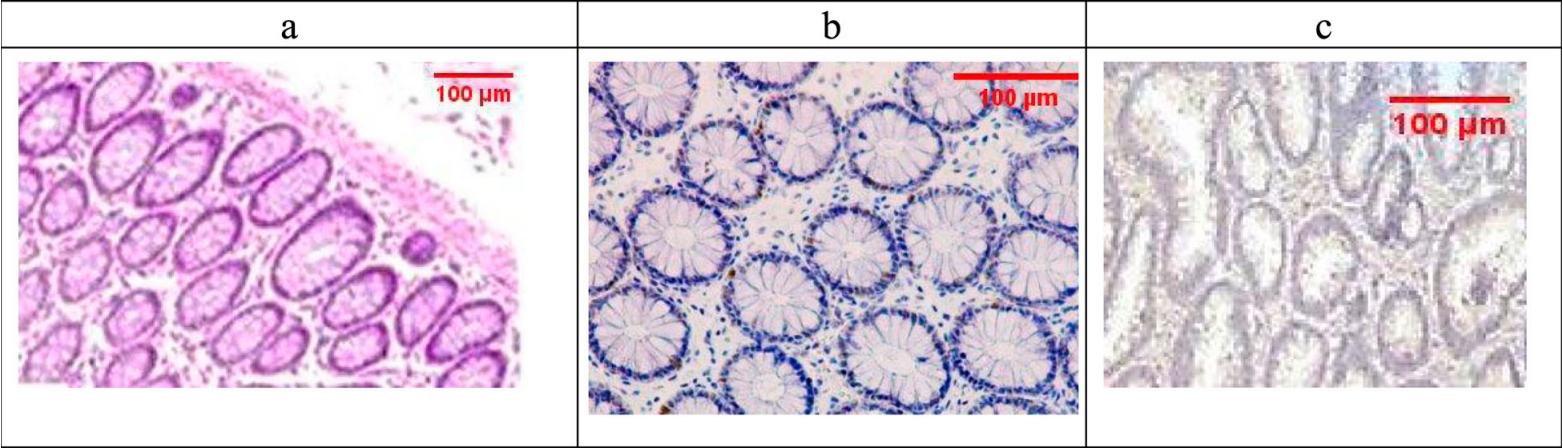
(a) Normal control colon specimen (H&E ×100), (b) normal colon stained by Bcl2 score as 1 (ABC ×100), (c): normal colon stained by Bax scored 1 (ABC ×100).

Examination of the untreated group showed that the lesion is well established with various tissue damage severity and various Geboes scorings, as demonstrated in Figures 3(a) and 4(a), which showed a marked increase in the neutrophils and many crypt abscesses with ulcerations compared with the normal colonic mucosa (Figure 5(a)).

**Figure 6.**
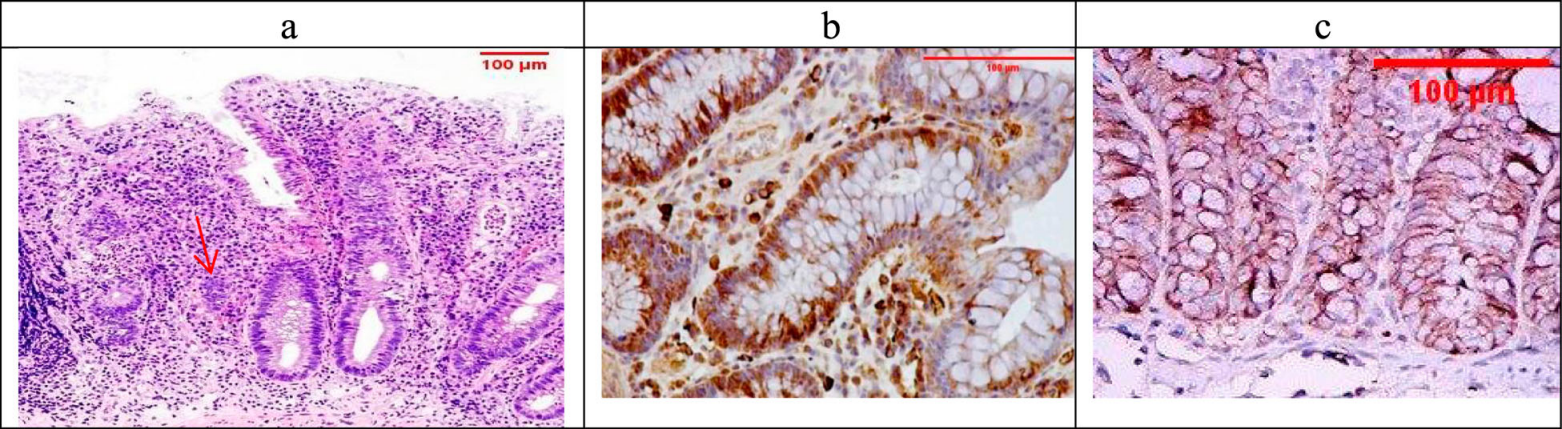
(a) The β-Glucan-treated group showing Geboes score 3.1 in the form of cryptitis (red arrow) and neutrophils seen in some of the epithelium (H&E ×100). (b) The same case showed a score 4 Bcl2 expression (ABC ×200), (c) also showed score a 3 core expression of Bax (ABC ×200).

**Figure 7.**
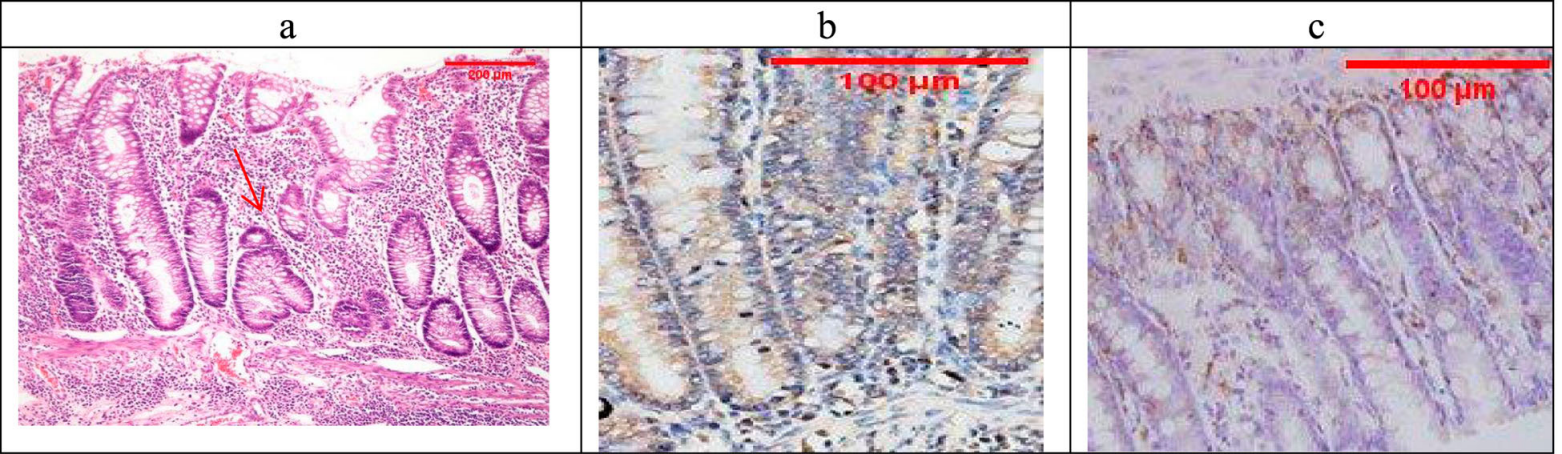
(a) The Celastrol-treated group showing Geboes score 3.2 in the form of cryptitis and neutrophils (red arrow) seen in most of the epithelium (H&E ×100). (b) The same case showed score a 2 Bcl2 expression (ABC ×200), (c) also showed a score 3 expression of Bax (ABC ×100).

Sections examined in the β-glucan-treated group showed that most of the cases showed UC cases with no severe cases (No Geboes score 4 at all) as seen in (Figure 6(a)), which showed moderate cryptitis, while the Celastrol-treated group also showed no severe cases of UC, but showed moderate cryptitis (No Geboes score 4), as seen in Figure 7(a).

**Figure 8.**
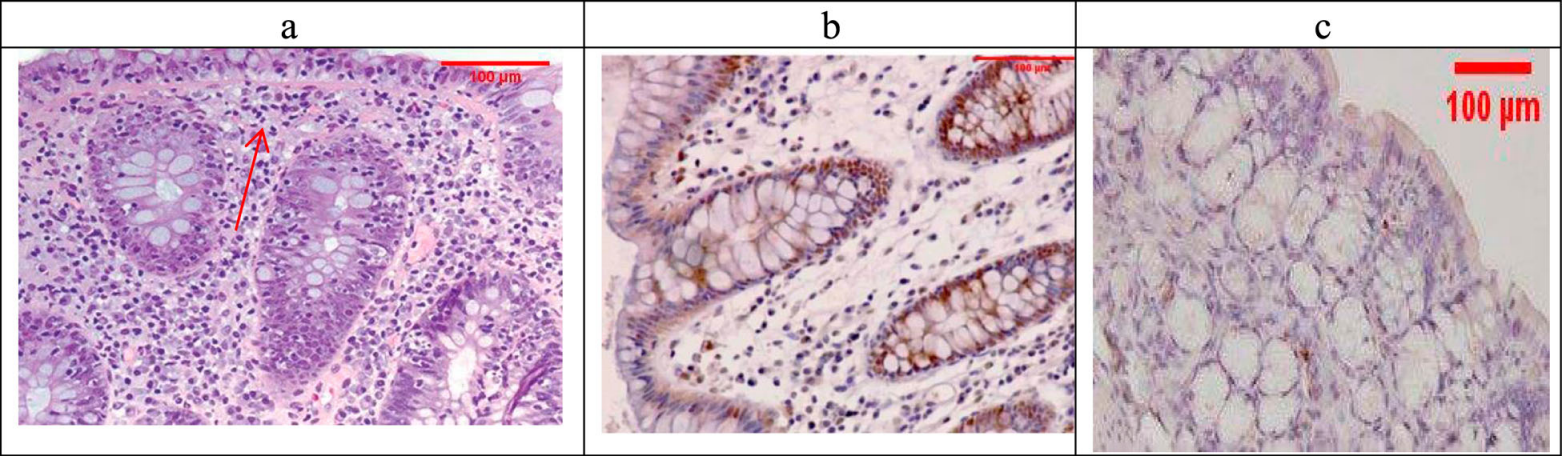
The combined-treated group showing Geboes score 1.2 in the form of a marked increase in the basal plasma cells only (red arrow) (H&E ×200). (b) The same case showed a score 2 Bcl2 expression (ABC ×200), (c) also showed a score 1 expression of Bax (ABC ×100).

The combined treatment group showed that all the samples were mild UC (No Geboes 3 nor 4), as seen in Figure 8(a), which showed increased plasma cells only with no neutrophils or cryptitis. Sections stained by Bcl2 and Bax to evaluate apoptosis seen in the control specimens showed that all the cases were score1 for both markers (Figure 5(b,c)).

For UC cases, as the Geboes score increases, the score c increases, as seen in Figures 3(b,c) and 4(b,c). Sections in the β-glucan treated group showed that both markers were still high score in high geboes scores, as seen in Figure 6(b,c), while in the Celastrol-treated group, most of the scores of both markers were low (Figure 7(b,c)) as there were no score 4 at all. In the combined treatment group, for all the UC cases, both markers were low expression (no score 3 nor 4 for both markers), as seen in Figure 8(b,c).

## 4. Discussion

There was a prodigious deal of research concerning the interrelation between gut microbiota imbalance and the occurrence of UC, highlighting the principal role of microbial intestinal flora in UC pathogenesis [6]. TMAO is a gut-derived toxic metabolite resulting from bacterial action on quaternary amines to release trimethylamine (TMA). The latter is consequently transformed into TMAO via flavin mono-oxygenase enzyme in the liver upon processing communal dietary constituents, such as choline [4]. Unlike other microbial metabolites that are swiftly metabolized and fail to reach readily detectable plasma concentrations, TMAO stays relatively stable over time, producing its toxic effects [28]. Thus, its concentration could reflect the extent of turbulence in the gut microbiome.

Remarkably, choline levels were significantly higher in IBD patients than normal individuals. Consequently, the level of TMAO is amplified in IBD and can act as a biomarker of disease presence and/r activity [28]. The raised TMAO concentrations are linked with systemic inflammation and amplified microbial dysbiosis in common variable immune-deficiency disease, signifying that TMAO could link dysbiosis and systemic inflammation. This could be accredited to the fact that TMAO enhances inflammatory markers expression by endorsing NF-κB initiation, and it could also prompt the release of other inflammatory cytokines [29]. These results came in harmony with our results that documented significantly raised TMAO levels in the UC group compared with the control group.

It is appealing to speculate that the tolerability and feasibility of oat β-glucan is theoretically efficient in depressing serum TMAO levels, as colon-derived toxin, in CKD patients [30]. Another finding stated that soluble fiber supplemented-diets could lessen TMA and TMAO metabolism by 40.6 and 62.6%, respectively. Moreover, dietary fibers alter gut microbial ecology and endorse beneficial bacterial species growth. It also improves the serum lipid profile, decreases energy intake and intestinal pH values. This description of microbial communities revealed some interesting associations, such as phyla that are negatively related to TMA and TMAO production [31]. These findings aligned with our findings that documented the modulating effect of β-glucan in lowering TMAO levels.

β-glucans fermentation by microbes results in the SCFAs, which are defined as saturated FA formed by one to six carbons of which acetic (C2), propionic (C3), and butyric (C4) are in the largest quantity [32]. They can be absorbed by the colonic epithelium supplying energy or playing a pertinent role in regulating the fatty acids metabolism. Moreover, an escalation in intestinal SCFA was perceived in animal models following interventions with oat β-glucans [33].

SCFAs acts as an activating agent to FFAR2/3 that are defined as a kind of the G-coupled protein receptors [34]; FFAR-2 is expressed in numerous tissues, and colon is one of them. SCFAs are the orthosteric ligand of FFAR2/3 as they bind to endogenous binding sites triggering covalent bonds between carbon atoms of SCAs and either two or one hydrogen atoms for interaction with FFAR-2 [35]. Therefore, in human and animal models, SCFAs are concomitant with FFAR-2 activation in regulating biological functions, such as hormonal synthesis [36], metabolic syndrome [37], and autoimmune disease occurrence [38].

The role of FFAR-2 in regulating intestinal inflammatory responses was previously investigated as FFAR-2-deficient mice have exacerbated disease symptoms in induced colitis as verified by reduced colon length, an amplified disease activity index and severe colonic inflammation; these results recommended that FFAR-2 arbitrates the protecting effects of SCFA in intestinal inflammation [39]. Likewise, studies concerning intestinal inflammation in animal models have proven the shielding effects of oral and intra-gastric β-glucan administration before or after chemical induction of colitis. This could be attributed to the ability of β-glucans to lessen pro-inflammatory markers expression in the colon, improving the clinical symptoms and protecting the gut from lesions, leukocyte infiltration and epithelial changes [33].

These findings aligned with our results ; we documented upregulation of FFAR-2 mRNA gene expression in β-glucan administrated groups compared to other groups explaining the triggering effects of β-glucans on FFAR-2 gene expression. Plausibly, concerning the modulation of gut microbio targeted , β-glucan managed to mend the dysbiosis-linked turbulence in UC by lowering TMAO level in β-glucans-treated groups. On the contrary, it upregulated FFAR-2 mRNA gene expression confirming the successful β-glucan effect in activating SCFA production with consequent FFAR-2 gene expression.

Furthermore, SCFAs play a key role in metabolism modifications and intestinal barrier function, such as mitochondrial ATP production and tight junction regulation in the epithelium of intestinal cells [40].

Although mitochondrial dysfunction plays in IBD is not fully elucidated, there is a substantial connection between intestinal inflammation and perturbed mitochondrial functions. Healthy mitochondria are important for the satisfactory energy supply needed for all cellular vital processes; thus, IBD patients are presented with reduced intestinal ATP levels, which can be taken as a marker for mitochondrial dysfunction [41]. Likewise, prevalent independent studies have stated that the most serious complication regarding mitochondrial function impairment is lessening ATP production, which directly results in intestinal epithelial cells apoptosis, as noticed herein. The damage caused by OS forces the mitochondria to enter a vicious cycle where the loss of respiration disturbs redox homeostasis resulting in increased ROS. Contemporary studies advised that enhancing and improving mitochondrial performance lessen the destructive effects of OS, such as inﬂammation and metabolic alterations [42].

Celastrol, the pentacyclic triterpenoid, has emerged as a therapeutic line acting to improve the mitochondrial dysfunction in a US model. It was demonstrated that in vitro Celastrol treatment led to mitochondrial function improvement as noticed by elevating intracellular ATP level, MMP level and CS activity in a palmitate-treated skeletal muscle [42]. These findings strengthen the emergent view that Celastrol could enhanced the OS status that took place as an inevitable consequence of mitochondrial dysfunction, which might be the cause of palmitate-arbitrated insulin resistance [43]. Moreover, Celastrol was able to hinder NF-κB and its downstream targets. It improved the oxidative metabolism of adipocytes by lessening NF-κB signaling pathways with subsequent mitochondrial functions improvement [44].

Furthermore, Celastrol repressed adipogenesis with metabolic disorder correction. This results in energy expenditure and mitochondrial gene upregulation. It also improved fatty liver disease by reducing lipid synthesis and elevating the anti-oxidative and anti-inﬂammatory status [45]. PGC-1α plays fundamental roles in mitochondrial genesis and respiration, and it was previously revealed its expression is reduced in UC and colon cancer patients. Moreover, it prevents colitis development and maintains the continuity of intestinal epithelium [46]. This reflects its imminent role in preserving mitochondrial function.

Celastrol administration surges the gene expression of PGC-1α in skeletal muscle and adipocytes in a diet-induced obese animal model that can endorse thermogenesis and white adipose tissues’ remodeling [47]. Celastrol also amplified the PGC-1α expression in skeletal muscles and adipocytes to lessen insulin resistance via AKT/P38 MAPK activation. It also repressed gluconeogenic activity by CREB/PGC-1α signaling mechanism. This research study considers the Celastrol stimulating effect on PGC-1α expression, promoting antioxidant enzymes that can protect intestinal cells against ROS and reduce the incidence of apoptosis, ameliorating the disease manifestations in IBD subjects [48]. PGC-1α activity is mandatory to regulate normal mitochondrial tasks, and in stressful conditions, PGC-1α is an operative stimulator of mitochondrial turnover and antioxidant effects [49]. Increased PGC-1α expression caused a noteworthy increase in antioxidant enzyme levels as catalase and SOD with subsequent prevention of OS with the reduction of apoptosis in intestinal cells.

## 5. Conclusion

By the insight gained, it was proved that the mutual use of β-glucan and Celastrol had novel curative effects in managing UC by targeting the Microbiota/Mitochondrial axis, paving for a new line for a promising intervention for such a complicated, multifaceted disease.

## 6. Limitations

To extrapolate our findings and to gain better mechanism deducing; *in vitro* experiments should be performed as a subsequent step to support data escalating and pave the way for further clinical application. Microbiota/Mitochondrial axis and its relation to UC must be further investigated in the upcoming research work.
